# Comparative and Evolutionary Genomics of Isolates Provide Insight into the Pathoadaptation of *Aeromonas*

**DOI:** 10.1093/gbe/evaa055

**Published:** 2020-03-20

**Authors:** Emilie Talagrand-Reboul, Sophie M Colston, Joerg Graf, Brigitte Lamy, Estelle Jumas-Bilak

**Affiliations:** e1 Équipe Pathogènes Hydriques Santé Environnements, UMR 5569 HSM, University of Montpellier, France; e2 Laboratoire de Bactériologie, Hôpitaux universitaires de Strasbourg, France; e3 US Naval Research Laboratory, National Academy of Sciences, National Research Council, Washington, District of Columbia; e4 Department of Molecular and Cell Biology, University of Connecticut; e5 Département de Bactériologie, CHU de Nice and Université Côte d’Azur, INSERM, C3M, Nice, France; e6 Département d’Hygiène Hospitalière, CHRU de Montpellier, France

**Keywords:** *Aeromonas*, opportunistic pathogens, genomes, pathogenomics, evolution, pathoadaptation

## Abstract

Aeromonads are ubiquitous aquatic bacteria that cause opportunistic infections in humans, but their pathogenesis remains poorly understood. A pathogenomic approach was undertaken to provide insights into the emergence and evolution of pathogenic traits in aeromonads. The genomes of 64 *Aeromonas* strains representative of the whole genus were analyzed to study the distribution, phylogeny, and synteny of the flanking sequences of 13 virulence-associated genes. The reconstructed evolutionary histories varied markedly depending on the gene analyzed and ranged from vertical evolution, which followed the core genome evolution (*alt* and *colAh*), to complex evolution, involving gene loss by insertion sequence-driven gene disruption, horizontal gene transfer, and paraphyly with some virulence genes associated with a phylogroup (*aer*, *ser*, and type 3 secretion system components) or no phylogroup (type 3 secretion system effectors, Ast, ExoA, and RtxA toxins). The general pathogenomic overview of aeromonads showed great complexity with diverse evolution modes and gene organization and uneven distribution of virulence genes in the genus; the results provided insights into aeromonad pathoadaptation or the ability of members of this group to emerge as pathogens. Finally, these findings suggest that aeromonad virulence-associated genes should be examined at the population level and that studies performed on type or model strains at the species level cannot be generalized to the whole species.

## Introduction

Aeromonads are ubiquitous Gram-negative bacilli primarily found in freshwater environments. The population structure of the genus *Aeromonas* has several characteristics that favor an evolutionary mode of species complexes that are heterogeneous groups of closely related but genetically distinct strains. These characteristics include a high rate of horizontal genetic transfer (HGT), a large genome harboring several ribosomal operons and a large pangenome encoding, in these bacteria, various metabolic capabilities supporting their adaptation to environmental changes and numerous virulence factors (Seshadri et al. 2006; Reith et al. 2008; Georgiades and Raoult 2011; Talagrand-Reboul et al. 2017). Moreover, aeromonads in the same aquatic habitat may be physically related, which allows sympatric speciation. In addition, this genus is a reservoir from which some species or subspecies may have emerged by allopatric speciation and specialized and/or adapted to particular niches, such as the specialized fish pathogen *Aeromonas salmonicida* subsp. *salmonicida* (Reith et al. 2008).

*Aeromonas* are emerging opportunistic pathogens often with an environment-to-human transmission route, resulting in a broad range of infections in humans (Janda and Abbott 2010; Khajanchi et al. 2010; Parker and Shaw 2011). Among the 30 validated species in the genus *Aeromonas*, 4 are most often associated with human diseases: *Aeromonas dhakensis*, *A. hydrophila*, *A. veronii*, and *A. caviae* (Janda and Abbott 2010; Chen et al. 2014; Wu et al. 2015). Additionally, aeromonads are able to colonize a wide range of animals, and some species, namely, *A. salmonicida*, *A. hydrophila*, *A. veronii*, *A. bestiarum*, and *A. piscicola*, are especially pathogenic to fish, causing septicemia and ulcerative and hemorrhagic diseases (Kozińska 2007; Janda and Abbott 2010; Beaz-Hidalgo and Figueras 2013).

As a characteristic of many opportunistic pathogens, the pathogenesis of *Aeromonas* infections is complex, multifactorial, and only partially elucidated to date. Aeromonads can express a wide repertoire of virulence factors involved in biofilm formation, cell adherence, invasion, and cytotoxicity, including polar and lateral flagella (Rabaan et al. 2001; Gavín et al. 2003), adhesins (Kirov et al. 1999), lipopolysaccharides (Canals et al. 2007), iron-binding systems (Byers et al. 1991; Massad et al. 1991), numerous extracellular toxins and enzymes (Braun et al. 2002) exported by different types of secretion systems (e.g., type 2 secretion system and type 3 secretion system [T3SS]) (Burr et al. 2002; Sha et al. 2005), and quorum-sensing systems (Swift et al. 1997, 1999; Kozlova et al. 2008; Khajanchi et al. 2012) that are critical for colonization (infection) and disease.

Whatever their provenance, *Aeromonas* spp. isolates harbor similar assemblages of virulence-associated genes (Chacón et al. 2003; Aguilera-Arreola et al. 2005), which has led to the presumption that exaptation occurred in environmental aeromonads (Cabello and Godfrey 2018), which facilitated host colonization, followed by pathoadaptation, which then resulted in adverse outcomes for the host. The elucidation of *Aeromonas* pathogenesis has been hampered by a number of impediments, such as an ambiguous correlation between virulence phenotypes and genetic content and low performance of tools for the detection of virulence-associated genes (Talagrand-Reboul et al. 2018). Conversely, current whole-genome sequencing approaches provide high-quality sequences to accurately study virulence genes and their genetic microenvironment.

In this context, we support that *Aeromonas* is an opportunistic bacterial pathogen characterized by various ecological niches, which can be considered “nurseries” suitable for genomic exchanges and rearrangements concerning genes involved in adaptation and virulence. This genomic flexibility, which is associated with the lifestyle of *Aeromonas*, may allow lineages to emerge that harbor, among others, capabilities of colonization and adhesion, in escaping innate immunity and in the production and secretion of toxins and exoenzymes. We hypothesized that the evolutionary process of virulence-associated genes will provide insight into pathoadaptation, and several questions were raised: Does the evolution of virulence genes follow the overall evolution of the genus? Are there any HGT of genes encoding virulence factors? Is the genetic microenvironment of these virulence genes conserved among strains? To provide the genomic basis of pathoadaptation in the genus *Aeromonas*, we studied a panel of virulence-associated genes from a large collection of genomes that reflect the current known diversity in the genus. We estimated the correlation between the genome-based phylogeny and the virulence-associated gene repertoire and used this information to address the questions raised and to form new hypotheses about the gain, maintenance, or loss of virulence factors throughout the genus *Aeromonas*.

## Materials and Methods

### Strains and Genomes

A total of 64 *Aeromonas* genomes were included in this study and are described in supplementary table S1, Supplementary Material online. They covered the 30 validated species and represented the type strain of every species or a reference strain in the case of *A. rivipollensis*, for which the genome of the type strain is not yet sequenced (supplementary table S1, Supplementary Material online). For the species with clinical relevance, we included several strains: *A. hydrophila* (*n* = 7), *A. dhakensis* (*n* = 6), *A. veronii* (*n* = 7), *A. caviae* (*n* = 7), *A. salmonicida* (*n* = 7), *A. rivipollensis* (*n* = 3), and *A. media* (*n* = 3). Whole genome sequencing was performed on four strains (this study), and 40 genomes were sequenced previously (Colston et al. 2014; Mosser et al. 2015; Talagrand-Reboul et al. 2018). The remaining 20 genomes were obtained from the public genome repository of NCBI (supplementary table S1, Supplementary Material online). Genome sequencing was performed at the Microbial Analysis, Resources and Services facility at the University of Connecticut (Storrs, USA) using an Illumina MiSeq benchtop sequencer after preparing libraries from the genomic DNA using a Nextera XT DNA sample preparation kit (Illumina, San Diego, CA). Paired-end reads were trimmed and assembled into scaffolded contigs using a de novo assembler of CLC Genomics Workbench version 6.1.5 (CLC-bio, Aarhus, Denmark) to obtain “improved high-quality draft genomes” (Chain et al. 2009). In all instances in which an isolate was reclassified as a different species (e.g., *A. hydrophila* subsp. *anaerogenes* CECT 4221, which was reclassified to the species *A. caviae*; Miñana-Galbis et al. 2013), we used the validated taxon to avoid any confusion. The draft genomes included are “high-quality draft genomes” (supplementary table S1, Supplementary Material online) based on sequencing and assembly metrics (e.g., average genome coverage and number of scaffolds) and verification of automated annotation (the presence of 15 housekeeping genes: *atpD*, *dnaJ*, *dnaK*, *dnaX*, *gltA*, *groL*, *gyrA*, *gyrB*, *metG*, *radA*, *recA*, *rpoB*, *rpoD*, *tsf*, and *zipA*).

### Genome Analysis

Complete and draft genomes were annotated using the RAST server to identify RNAs and protein-coding genes (Overbeek et al. 2014). Based on the quality metrics, all the genomes included in this study were sufficient for the assessment of virulence-associated gene content and the comparison between strains (supplementary table S1, Supplementary Material online) (Chain et al. 2009). The genomes were screened for genes encoding virulence factors acting by various mechanisms (toxins, enzymes, secretion system components, and flagellin) and well characterized in *Aeromonas* spp. (table 1) by using reference protein sequences and either translated sequences of the validated subset of UniProt (SwissProt) or annotated genes of the previously sequenced *Aeromonas* spp*.* in the TrEMBL database. Sequence comparisons with reference protein sequences were performed with SEED viewer, which uses bidirectional protein–protein BLAST (BlastP) sequence comparison of translated open reading frames. Proteins with amino acid sequence similarities ≥65% and *E*-values ≤10^−10^ were considered homologs (Altschul and Lipman 1990). All the results of BlastP analysis were manually verified. Sequences homologous to virulence-associated genes were checked to identify coding sequences (CDSs) that harbor an open reading frame without nonsense mutations. The HMMSCAN program (HMMER website, EMBL-EBI, Potter et al. 2018) was used to evaluate the putative impact of nucleotide polymorphisms between homologs on protein functions. Amino acids in aerolysin that are critical for oligomerization (H in 155) and heptamerization (K in 374 and E in 390) were examined in the protein homologs (Degiacomi et al. 2013). The neighboring CDS order of the considered loci, or “flanking gene organization,” was qualitatively compared among the different strains on the basis of RAST annotation when genes were not at the end of a contig or interrupted by contig gaps. The analyzed region on each side of the genes included 1) the right and left directly flanking coding DNA sequences, 2) an extended region up to three CDS positions if these configurations were conserved in the majority of strains, and 3) a more extended region that would be gathered with one flanking gene found in another close position, up to seven CDSs. To detect putative HGT, we manually compared the relative branching order of the 13 virulence gene phylogenies within each phylogroup and within each species when several strains had been included. Mobile genetic elements (MGEs) were searched by using ISsaga2 (Mobile Genetic Elements team- CNRS, UMR5100, Toulouse, France) to detect insertion sequences (ISs) and ICEfinder (Microbial Bioinformatics Group, Shanghai, China) to detect integrative and mobilizable elements (IMEs) and integrative and conjugative elements (ICEs). We manually verified whether virulence genes were located in the predicted IME/ICE regions.

**Table 1:** Aeromonad Virulence Factors Studied

Virulence Factor	Reference Sequences			Virulence-Associated Gene	Genomic Location	Source
	Accession No.	Strain	Length (Amino Acids)			
Aerolysin AerA (syn: Cytolytic enterotoxin Act)	P09167^S^ (AerA) Q44063^E^ (Act)	*bestiarum Ah65* *A. dhakensis* SSU </>	493493	*aer* (syn: *act*)	Chromosome	Howard et al. (1987), Chakraborty et al. (1987), Chopra et al. (1993)
Thermolabile cytotonic enterotoxin	Q44061^E^	*A. dhakensis* SSU	368	*alt*	Chromosome	Chopra et al. (1996)
Thermostable cytotonic enterotoxin	Q8VRN3^E^	*A. dhakensis* SSU	636	*ast*	Chromosome	Sha et al. (2002)
Extracellular collagenase	J7FWV3^E^	*A. piscicola* AH-3	915	*colAh*	Chromosome	Duarte et al. (2015)
Toxin RtxA (repeat-in-toxin A)	A0KHZ7^E^	*A. hydrophila* subsp. *hydrophila* ATCC 7966^T^	4,685	*rtxA*	Chromosome	Suarez et al. (2012)
Exotoxin A	A0A0W0AX19^E^	*A. salmonicida* Y577	639	exoA	Unknown (chromosome in *P. aeruginosa* PA01)	Tsaur and Clowes (1989), Ponnusamy et al. (2016), Vincent et al. (2016)
SST3 needle protein AscF	Q6WG33^E^	*A. veronii* 283c	85	*ascF*	Chromosome or plasmid (chromosome in *A. hydrophila* ANNIH1 and ALO6-06/ plasmid in *A. salmonicida* subsp. *salmonicida* A449)	Chacon et al. (2004), Reith et al. (2008); Tekedar et al. (2015),Hughes et al. (2016)
SST3 component AscG	Q6WG32^E^	*A. veronii* 283c	116	*ascG*		
SST3 Inner membrane channel protein AscV	A4SUH2^E^	*A. salmonicida* subsp. *salmonicida* A449	721	*ascV*		
ADP-ribosyltransferase toxin AexT	Q93Q17^S^	*A. salmonicida* subsp. *salmonicida* A449	475	*aexT*	Chromosome	Stuber et al. (2003), Dacanay et al. (2006), Reith et al. (2008), Silver and Graf (2009)
ADP-ribosyltransferase toxin AexU	D5LUP3^E^	*A. veronii* bv. *sobria* AeG1	512	*aexU*	Chromosome	Sha et al. (2007), Silver and Graf (2009), Abolghait et al. (2011)
Lateral flagellin A	Q93TL9^E^	*A. caviae* Sch3	281	*lafA*	Chromosome	Kirov et al. (2002), Canals et al. (2006)
Serine protease Ahe2	A4SNU7^E^	*A. salmonicida* subsp. *salmonicida* A449	625	*ser* (syn.: *ahe2*)	Chromosome	Reith et al. (2008)

Note.—S/E, accession numbers correspond to protein sequences in SwissProt or TrEMBL databases.

**Table 2:** Summary of Targeted Virulence Gene Analysis Results in Aeromonad Genomes

Virulence-Associated Genes	RAST Annotation of Coding DNA Sequence (Length in Amino acids, AA)	% of Presence (%)	Present	Absent	Flanking Sequences	
					Upstream	Downstream
*aer/act*	Hemolysin (481–505 AA)	52	Most phylogroups	Caviae and Media phylogroups	Hypothetical protein	Mostly hydroxymethyl pyrimidine phosphate synthase ThiC
*alt*	Putative lipase (791–819 AA)	97	Most phylogroups	*A. fluvialis* and *A. lacus*	Hypothetical protein	GlpG except in the Schubertii phylogroup
*ast*	Predicted exported alpha-*N*-acetylgalactosaminidase (628–637 AA)	19	Hydrophila, Salmonicida, and Veronii phylogroups	Most phylogroups	Putative pyridoxine 5′-phosphate synthase (or hypothetical protein or a putative aspartate amino transferase)	Putative tagatose 1,6 bi-phosphate aldolase
*colAh*	Microbial collagenase secreted (910–920 AA)	89	Most phylogroups	Variable	Putative *O*-succinyl acid-CoA ligase (or hypothetical protein)	SanA protein
*rtxA*	RtxA toxin (4439–4849 AA)	14	Hydrophila phylogroup	Most phylogroups	RTX toxin activating lysine acyltransferase	Variable
exoA	Putative exotoxin A precursor (639–640 AA)	14	< id="625" data-dummy="list" list-type="suimple"> Hydrophila phylogroup A. piscicola </>	Most phylogroups	Putative cyanate transporter protein CynX	Ribosomal large subunit pseudouridine synthase F
*ascF*	Cytoplasmic protein AscF (82–89 AA)	33	Most phylogroups	Caviae, Media, and Molluscorum phylogroups	AscE	AscG
*ascG*	AscG (114–118 AA)				AscF	AscH
*ascV*	AscV T3SS inner membrane channel (705–706 AA)				AscY chaperone protein	Variable
*aexT*	ADP-ribosyltransferase (451–476 AA)	13 (38% of T3SS+ genomes)	*A. veronii* and *A. salmonicida*	Most phylogroups	Type 3 secretion chaperone protein	AexU in *A. veronii* and EAL domain
*aexU*	ADP-ribosyltransferase (446–514 AA)	20 (62% of T3SS+ genomes)	Encheleia, Salmonicida, Hydrophila, and Veronii phylogroups	Schubertii, Molluscorum, Media, and Caviae phylogroups	Type 3 secretion chaperone protein (or AexT)	EAL domain or phenylalanine tRNA synthetase beta subunit
*lafA*	LafA flagellin protein (280–297 AA)	50	Most phylogroups	Variable	Variable	Putative LafB flagellar hook-associated protein
*ser/ahe2*	Putative extracellular protease (624–634 AA)	67	Most phylogroups	< id="692" data-dummy="list" list-type="suimple"> Schubertii and Caviae phylogroups A. media </>	Variable	Hypothetical protein

### Genome-Based Phylogenetic Relationships

All genome assemblies used in this study were adequate to reconstruct a single-nucleotide polymorphism (SNP)-based phylogenomic tree using the k-mer method. The aeromonad phylogeny was inferred using the kSNP 3.1 software package in which SNPs were based on k-mer analysis (Gardner et al. 2015). The maximum-likelihood (ML) tree was reconstructed on the basis of 27,856 SNPs identified in whole-genome sequences (WGS) for at least 75% of all strains (k-mer = 19). The aligned SNP sequences were used for decomposition analyses with the neighbor-net algorithm available in SplitsTree 4.0 software (Huson and Bryant 2006).

### Phylogenetic Analysis of Virulence-Associated Genes

Phylogeny was inferred from the sequences of 13 virulence-associated genes (*aer*/*act*, *alt*, *ast*, *colAh*, *rtxA*, *exoA*, *ascF*, *ascG*, *ascV*, *aexT*, *aexU*, *lafA*, and *ser*). In the case of T3SS genes (*ascF*, *ascG*, and *ascV*), the three genes were concatenated. Nucleotide sequences were aligned using the Clustal ω2 program in the Seaview 4 package (Gouy et al. 2010). ML phylogenetic trees were reconstructed for each gene or concatenated genes using the best-fit model of evolution determined by the Akaike criterion (http://iqtree.cibiv.univie.ac.at/). ML bootstrap supports were calculated after 100 reiterations. To study the phylogenetic inference of the *exoA* gene, we added the nucleotide sequence of the *eta* gene (GenBank locus PA1148) coding for exotoxin A (SwissProt PA11439) produced by *Pseudomonas aeruginosa* PA01.

### Statistics

All qualitative variables were compared using a *χ*^2^ test, and all quantitative variables were compared using Student’s *t*-test, wherein a *P* value ≤0.05 was considered significant. All these computations were performed using R project software (http://www.r-project.org).

## Results

### Phylogenomic Relationships in Aeromonads

**Fig. 1.**
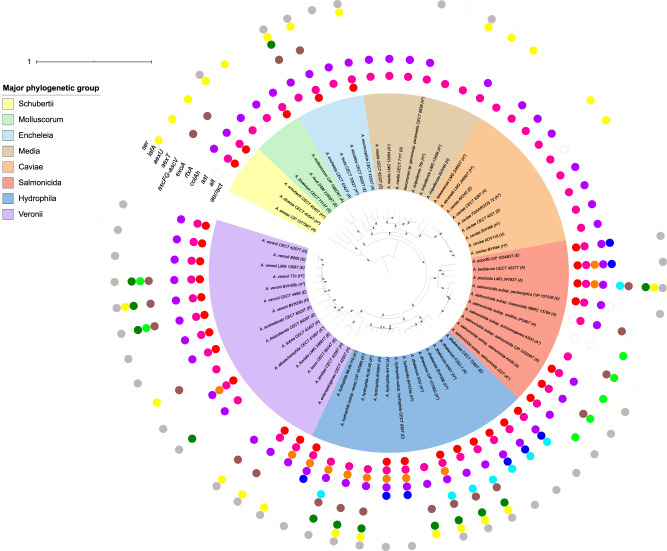
—ML phylogeny based on 27,856 SNPs and the virulence-associated gene repertoire. The tree shows the phylogeny of 64 *Aeromonas* strains, including the 30 validated species represented by their type strain or a reference strain (*A. rivipollensis*). The scale bar is expressed as changes per total number of SNPs. The numbers at the nodes are support values estimated with 100 bootstrap replicates. Only bootstrap values ≥70 were indicated. Eight well-supported clades named “Major phylogenetic groups” are shown by colored ranges on strain labels: group Schubertii, group Molluscorum, group Encheleia, group Media, group Caviae, group Salmonicida, group Hydrophila, and group Veronii. The isolation source is indicated in parentheses after the strain number: environmental (E), animal (A), or human (H). An asterisk denotes whether pathogenic phenotypes have been described for the strain. The external colored circles corresponded to genes that encoded virulence factors detected after genome BLAST analysis, including from the inside to the outside: *aer/act* for a toxin with two denominations, “Aerolysin” or “cytolytic enterotoxin Act”; *alt* for a thermolabile cytotonic enterotoxin Alt; *ast* for a thermostable cytotonic enterotoxin Ast; *colAh* for an extracellular collagenase ColAh; *rtxA* for a repeat-in-the-toxin A; *exoA* for an exotoxin A, *ascF*, *ascG*, and *ascV* for T3SS components; *aexT* for an ADP-ribosylating transferase and T3SS-effector AexT; *aexU* for an ADP-ribosylating transferase and T3SS-effector AexU; *lafA* for a lateral flagellin A LafA; and *ser* for an extracellular serine protease Ahe2/AspA. Open circles indicate interrupted genes.

A phylogenetic ML tree based on SNPs present in 75% of the strains provided information on the relative phylogenetic placement of the studied strains (27,856 SNPs; fig. 1). This SNP-based phylogenomic approach led to the reconstruction of a robust tree that delineated the eight major phylogenetic groups (bootstraps = 100) previously clustered by core-based phylogenomics or multilocus phylogeny based on 15 housekeeping genes (Colston et al. 2014), hereafter called phylogroups Schubertii, Hydrophila, Veronii, Caviae, Media, Encheleia, Salmonicida, and Molluscorum. Every taxonomical species represented by several strains was distinctly separated from the others in the phylogenomic ML tree (bootstraps = 100).

The phylogenetic network generated by the neighbor-net analysis (supplementary fig. A1, Supplementary Material online) was overall congruent with the phylogenomic ML tree. In addition, it showed interconnections between phylogroups, species within phylogroups and strains within species. Such reticulations suggest recombination events (e.g., horizontal gene transfers) that may have taken place during evolution. Recent events obviously occurred among the *A. salmonicida* lineages, with the exception of the mesophilic strain (supplementary fig. A1, Supplementary Material online).

### Repertoire and Distribution of Virulence Genes in Aeromonads

The characteristics and annotations of the targeted virulence genes as well as the flanking regions are summarized in table 2. The results of genome screening for the presence of 13 virulence-associated genes are shown for every genome in the phylogenomic ML tree (fig. 1). Virulence-associated genes were distributed unevenly among the phylogroups, from “no gene” in *A. fluvialis* LMG 24681^T^ to “nine genes out of ten” in *A. dhakensis* SSU and *A. piscicola* LMG 24783^T^. The strains belonging to the phylogroups Hydrophila and Salmonicida contained significantly more virulence-associated genes (means of 6.8 and 5.9 genes detected from the test panel, respectively) than those from the phylogroups Media and Caviae (means of 2.2 and 2.6 genes detected, respectively; *P* value <0.001).

Each virulence gene fell into one of three categories based on its distribution among genomes (fig. 1 and table 2). The genes of the first group (*alt*, *colAh*, and *lafA*) were well represented in the whole tree except in some scattered genomes. The genes of the second group (*aer*/*act* and *ser*) were widely found among the tree, except in some specific phylogroups, for example, the Caviae and Media groups. In addition, a significant association between the presence/absence of the *aer* and *ser* genes was noted with 29 *aer*+/*ser*+ and 17 *aer*−/*ser*− strains out of the 64 genomes (*P* value <0.001). The genes of the third group (*ast*, *rtxA*, *exoA*, *ascF*, *ascG*, *ascV*, *aexT*, and *aexU*) were more specifically associated with particular genomes or phylogroups. For instance, the distributions of the genes *rtxA* and *exoA* were restricted to the phylogroup Hydrophila (*A. dhakensis* and *A. hydrophila*) and to the mesophilic species in the phylogroup Salmonicida, that is, *A. salmonicida* subsp. *pectinolytica* (fig. 1). None of the virulence-associated genes were present in any of the genomes.

The HMMSCAN program detected a peptide signal in each CDS homologous to the aerolysin, collagenase, Ast, exotoxin A, and serine protease genes. In addition, all aerolysin homolog genes were predicted to encode APT and aerolysin domains. The amino acid positions important for oligomerization (H in 155) and heptamerization (K in 374 and E in 390) were conserved with the exception of a change of unknown consequence with N instead of K at position 374 in the aerolysin gene of *A. tecta* CECT 7082T. The N-terminus of a bacterial virulence factor lipase and a peptidase M9 domain were detected in all the *alt* and *colAh* genes, respectively. The RtxA CDSs were predicted to have 28–30 RtxA repeats, 2–3 coiled coils, an actin-linking domain (except *A. bestiarum* and *A. popoffii* strains), a yersinia-like virulence antigen (except *A. hydrophila* ML09-119), a serine aminopeptidase S33a domain or an alpha/beta hydrolase family domain, a peptidase C80 domain with 2 predicted active sites, and a membrane localization domain (except *A. hydrophila* ML09-119). All the exotoxin A genes harbored exoA-binding, exoA-targeting, and exoA-catalytic domains with one predicted active site. The T3SS structural genes were predicted to carry their appropriate functional domains. The ADP-ribosylating transferase toxin genes *aexT* and *aexU* shared one YopE domain when all the *aexT* genes also harbored one ADP-ribosyltransferase exoenzyme domain with two predicted active sites. All the flagellar *lafA* homologs had bacterial flagellin N-terminal and C-terminal helical regions. A peptidase S8 family protein with three predicted active sites and a proprotein convertase P-domain were detected in every serine protease gene. Overall, this predictive analysis supports the presumption that the homologs studied herein may be able to generate functional proteins after the appropriate transcription, translation, and posttranslation modifications.

In most species for which multiple genomes were assessed, there was no obvious relationship between the isolate origin and the virulence gene content. For instance, *A. hydrophila* and *A. dhakensis* recovered from the environment and animals (CECT 839 and ML09-119) or humans (CECT 7289 and AAK1) exhibited identical profiles that included the aerolysin gene. Similar observations were made in the species *A. veronii*, *A. media*, and *A. caviae* (fig. 1). An exception was *A. dhakensis*, for which only three strains recovered from human disease cases harbored the genes for the T3SS apparatus.

In addition to the 340 virulence gene homologs detected without nonsense mutations, RAST identified 19 ISs that interrupted several virulence-associated genes except *ascF*, *ascG*, *ascV*, and *aexU*, which were not interrupted (fig. 1). Eight of the ISs were located at the ends of contigs; therefore, their annotations remain putative (data not shown). Twelve of the 19 detected ISs (63%) were recovered from *A. salmonicida* genomes, representing 11% of the studied genomes; this result is consistent with the systematic search of MGEs performed in genomes (supplementary table S1, Supplementary Material online) because the highest numbers of putative ISs were found in the species *A. popoffii*, *A. allosacharophila*, and *A. salmonicida*, with 31, 41, and from 17 to 432 ISs detected, respectively. More generally, ISs were predicted in all the genomes analyzed (min. of 1, max. of 432, median of 15 ISs). IMEs and/or ICEs were predicted in 47% of the strains and belonged to all eight major phylogroups. From one to two IMEs were detected in 38% of the strains, of which no directed repeats were identified for 29%. We observed a genomic colocalization between MGEs and virulence genes only for *alt* in *A. rivipollensis* 76c and for *colAh* in *A. salmonicida* subsp. *smithia*.

### Phylogeny of Virulence-Associated Genes

The ML phylogenetic trees of the ten targeted virulence genes or gene combinations were reconstructed and compared with the structure of the phylogenomic tree. The content and synteny of the regions flanking the virulence genes were also considered to reconstruct evolution hypotheses of virulence genes in the genus *Aeromonas*.

#### *Core Genome-like Evolution of the* alt, colAh, *and* ser *Virulence Genes*

**Fig. 2.**
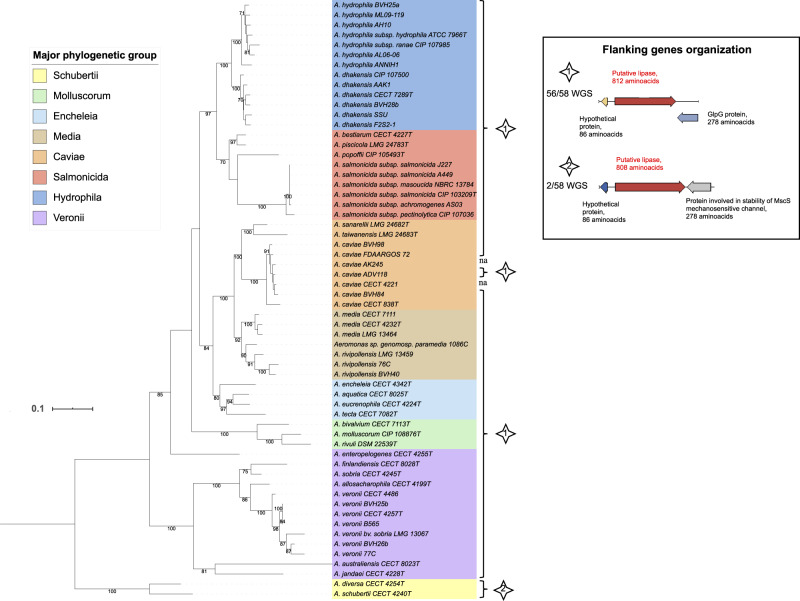
—ML tree based on *alt* gene sequences (2,526 nt) reconstructed using the TIM3 model plus gamma distribution and invariant sites as a substitution model with 60 complete nucleotide sequences. The two interrupted sequences (*A. simiae* and *A. salmonicida* subsp. *smithia* homologs of *alt*) are not represented in this tree. The horizontal lines represent genetic distance, with the scale bar indicating the number of substitutions per nucleotide position. The numbers at the nodes are support values estimated with 100 bootstrap replicates. Only bootstrap values ≥70 are indicated. The major phylogenetic group of each strain is indicated by colored ranges on strain labels. The type of genetic organization of the flanking genes shown in the inserted box is indicated for each strain with a numbered star. Abbreviations: na, not applicable; WGS, whole-genome sequences.

The gene *alt* was detected in all studied genomes except *A. fluvialis* LMG 24681^T^ and *A. lacus* CECT 8024^T^ (i.e., 97% of the genomes). The *alt* ML tree shown in figure 2 is emblematic of congruence between virulence-associated gene phylogeny and phylogenomics. Indeed, *alt* gene sequences from the eight phylogroups clustered in eight clades (bootstrap ≥70), in which the sequences from the species *A. hydrophila*, *A. dhakensis*, *A. rivipollensis*, *A. media*, *A. veronii*, and *A. salmonicida* were well separated (bootstrap ≥91). The region flanking the *alt* gene encoded the same putative GlpG protein in all genomes except in the Schubertii genomes that encoded a putative protein involved in the stability of the MscS mechanosensitive channel in place of GlpG.

Similar to *alt*, *colAh* was widely distributed in all but seven genomes (i.e., 89% of the genomes) and scattered among phylogroups. The *colAh* phylogeny (supplementary fig. A2, Supplementary Material online) was also congruent with the phylogenomics with the sole exception of *A. bivalvium* CECT 7113^T^, whose *colAh* sequence clustered with the group Salmonicida. In addition, the species *A. caviae*, *A. hydrophila*, *A. dhakensis*, and *A. salmonicida* were robustly delineated by the *colAh* phylogeny (bootstrap ≥96). The two *colAh*-flanking regions were clade specific with a putative *O*-succinyl acid-CoA ligase in the phylogroups Media, Encheleia, Caviae, and Molluscorum; a hypothetical protein in the groups Hydrophila and Salmonicida; or one or the other one in the groups Veronii and Schubertii.

The gene *ser* was present in only 67% of the genomes but clearly demarcated (bootstraps = 100) five groups, Veronii, Hydrophila, Salmonicida, Media, and Molluscorum, within which the sequences from the species *A. veronii*, *A. hydrophila*, *A. dhakensis*, *A. rivipollensis*, and *A. salmonicida* were well separated (bootstrap >70) (supplementary fig. A3, Supplementary Material online). The *ser* gene was absent from the Caviae and Schubertii groups and only detected sporadically in the Media phylogroup, where *ser* was present in *A. rivipollensis* (3/3), and in the strain *Aeromonas* sp. genomospecies paramedia 1086C but was absent from the three *A. media* sensu stricto strains (fig. 1). Eight different *ser*-flanking regions displayed an overall but partial superposition with phylogroups. In addition to this vertical evolution backbone, some signals of recombination were detected. The group Encheleia was paraphyletic (bootstraps = 98) in *ser* phylogeny with two different *ser*-flanking regions in the two Encheleia clades. In addition, *A. bivalvium* CECT 7113^T^ grouped with *A. rivipollensis* in *ser* phylogeny. These findings suggest that *ser* genes display a core genome-like evolution (ancestral and vertical inheritance) with some significant MGE transfer events between bacteria or “horizontal gene transfers” (HGT).

In conclusion, the prevalence of *alt* and *colAh* genes within the genus, the congruence of virulence gene-based trees and phylogenomics, and the conservation of genes and flanking regions according to clades all suggest a common ancestral inheritance of *alt* and *colAh* genes and that their evolution was directly linked to that of the core genome and to speciation in the genus. The backbone of *ser* phylogeny also displayed a core genome-like evolution, but the absence of complete aerolysin-Ser systems in several phylotypes, species, or strains suggests the loss of *ser*, collectively or independently. Recombination events or HGT were not detected in *alt* phylogeny, were scarce in *colAh*, and were more apparent in *ser* phylogeny.

#### Paraphyly of Aerolysin in Aeromonads

**Fig. 3.**
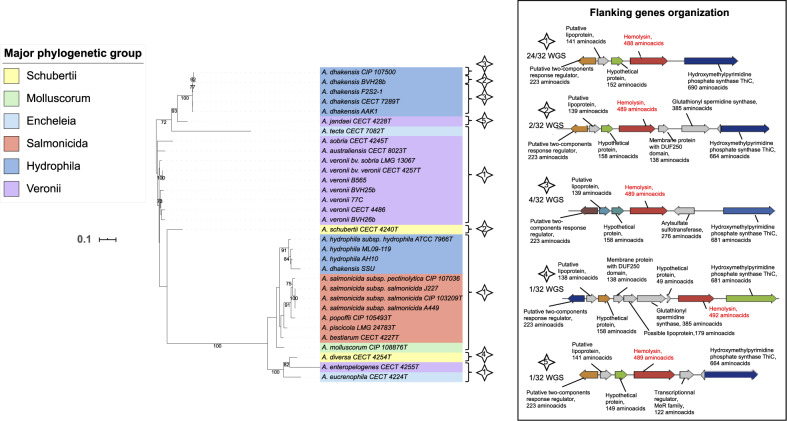
—ML tree based on *aer/act* gene sequences (1,527 nt) reconstructed using the TIM model plus gamma distribution as a substitution model from the 32 complete nucleotide sequences. One interrupted sequence (*A. salmonicida* subsp. *masoucida* homolog of *aer*/*act*) is not represented in this tree. The horizontal lines represent genetic distance, with the scale bar indicating the number of substitutions per nucleotide position. The numbers at the nodes are support values estimated with 100 bootstrap replicates. Only bootstrap values ≥70 are indicated. The major phylogenetic group of each strain is indicated by colored ranges on strain labels. The type of genetic organization of the flanking genes shown in the inserted box is indicated for each strain with a numbered star. Abbreviation: WGS, whole-genome sequences.

The *aer/act* gene was present in 52% of the evaluated genomes. The ML phylogeny for *aer/act* delineated two main clades (bootstrap = 100), where each clade contains members from phylogroups Schubertii, Hydrophila, Veronii, and Encheleia but not from phylogroups Caviae and Media (fig. 3). The distribution within the two clades was dependent on species rather than phylogroup. For instance, the *aer/act* sequences from *A. veronii* and *A. dhakensis* (group Hydrophila) strains (except the strain SSU) belonged in one clade, whereas all the sequences from *A. hydrophila* (group Hydrophila) and *A. salmonicida* were in the other clade. The aerolysin genes in aeromonads were split into two clades without phylogenomic congruence. For instance, the homologs in *A. dhakensis* SSU and *A. dhakensis* BVH28b shared only 66% sequence identity (78% similarity) with proteins with lengths of 504 and 488 amino acids, respectively. However, the two types of aerolysin genes were clearly homologous and shared the same microenvironment (fig. 3). The most likely hypothesis for aerolysin evolution is that two *Aeromonas* ancestors each independently acquired the *aer*/*act* variants and that they were maintained in the different phylogroups. The near species-specific distribution of the two types of *aer*/*act* genes is possibly further evidence for their putative involvement in the speciation process that occurred later in aeromonads.

#### Complex Evolution of Lateral Flagellin among Aeromonads

**Fig. 4.**
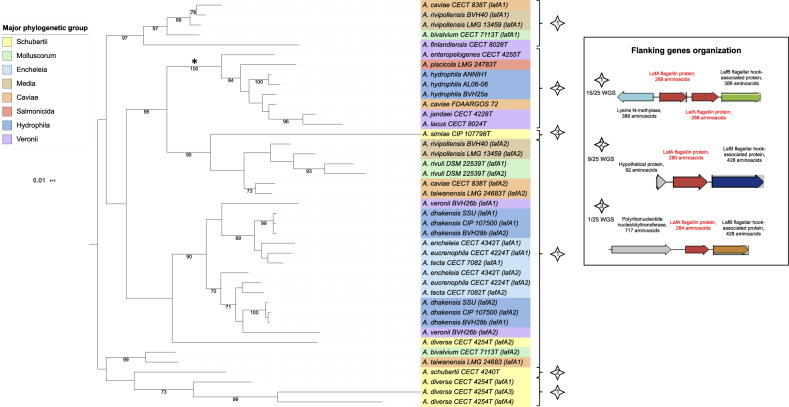
—ML tree based on *lafA* gene sequences (906 nt) reconstructed using the TIM model plus gamma distribution and invariant sites as a substitution model from 41 complete *lafA* nucleotide sequences. Each different copy found in a genome (or a strain) was arbitrarily numbered (*lafA*x). The same *lafA* numbering but in two different species corresponded to different *lafA* sequences. The clade gathering most of the genomes with a monocopy of *lafA* is indicated by an asterisk (*). The numbers at the nodes are support values estimated with 100 bootstrap replicates. Only bootstrap values ≥70 are indicated. The major phylogenetic group of each strain is indicated by colored ranges on strain labels. The type of genetic organization of the flanking genes shown in the inserted box is indicated for each strain with a numbered star. Abbreviation: WGS, whole-genome sequences.

Multiple copies of the *lafA* gene, which encodes lateral flagellin, were found in several genomes: 2 different copies in 13 genomes and 4 different copies in the genome of *A. diversa* CECT 4254^T^. Within the Hydrophila phylogroup, *A. hydrophila* strains (*n* = 3) had a unique copy, whereas the three strains of *A. dhakensis* harbored two different copies. The *lafA* ML tree was structured in four clades (bootstrap ≥ 90) and several weakly supported lineages (fig. 4). The *lafA* genes from the genomes that contained only one copy were mostly grouped in one clade (asterisk in fig. 4) except for the *A. finlandiensis*, *A. simiae*, and *A. schubertii* strains. Duplicated *lafA* genes formed another clade, which further separated into two subclades (bootstrap = 70) that encompass all strains of the phylogroup Encheleia and of the species *A. dhakensis*. In all cases, a CDS encoding a putative LafB flagellar hook-associated protein was located downstream (fig. 4). The prevalence of *lafA* within the genus *Aeromonas* (all the major phylogroups), the locus organization, and its phylogenetic inference suggest a common ancestral inheritance and that duplication occurred before mutations, insertions, or loss events during the evolutionary history of the genus. Despite ancestral inheritance, the high level of incongruence between the *lafA* phylogeny and phylogenomics suggests frequent HGT events in aeromonads (fig. 4 and table 2).

#### HGT Drive the Evolution of TSS3, TSS3 Effectors, and Toxin-Encoding Genes in Aeromonads

**Fig. 5.**
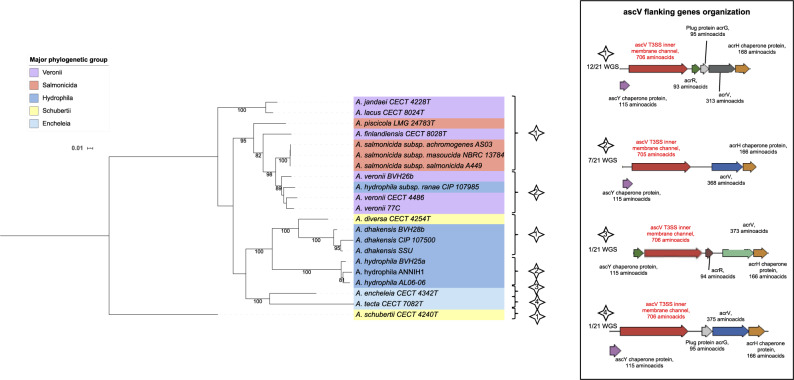
—ML tree based on concatenated sequences of the T3SS coding gene sequences *ascF*, *ascG*, and *ascV* (2,739 nt) reconstructed using a GTR model plus gamma distribution as a substitution model from 21 complete nucleotide sequences. The numbers at the nodes are support values estimated with 100 bootstrap replicates. Only bootstrap values ≥70 are indicated. The major phylogenetic group of each strain is indicated by colored ranges on strain labels. The type of genetic organization of the flanking genes shown in the inserted box is indicated for each strain with a numbered star. Abbreviation: WGS, whole-genome sequences.

The *ascF*, *ascG*, and *ascV* genes, which encode the structural components of the T3SS, were never detected separately. Therefore, they have been concatenated for further analysis (*ascFGV*). They were detected in 21 studied genomes (i.e., 33% of the genomes) covering all the major phylogenetic groups, except the groups Caviae, Media, and Molluscorum (fig. 1). The ML tree of the concatenated sequences showed four major clades (bootstraps ≥ 95; fig. 5), corresponding to the Salmonicida/Veronii, Hydrophila, Encheleia, and Veronii groups. The strain *A. schubertii* represented an external branch, and the strains *A. hydrophila* subsp. *ranae* and *A. diversa* belonged to the Salmonicida/Veronii and Hydrophila *ascFGV* clades, respectively. From a functional point of view, the presence of *ascFGV* was associated with at least one T3SS effector gene (*P* value <0.001). The T3SS effectors *aexT* and *aexU* were absent from six T3SS-encoding genomes (29%). Conversely, in four of the *aexU-* and/or *aexT*-positive genomes (21%), no T3SS component-encoding genes were detected.

Concerning T3SS effectors, the gene *aexT* was present in five *A. salmonicida* and two *A. veronii* strains and was found in the same locus as the gene *aexU* only in the *A. veronii* strains. The *aexU* gene was detected from the genomes of 13 strains belonging to the species *A. hydrophila*, *A. dhakensis*, *A. veronii*, *A. allosacharophila*, *A. piscicola*, and *A. encheleia* (fig. 1). The *aexT* ML tree showed two clades that aggregate *A. veronii* strains and *A. salmonicida* strains and that harbor a specific genetic locus organization (numbered 1 and 2 in supplementary fig. A4, Supplementary Material online). Four organizations of *aexU*-flanking genes were revealed for the locus (supplementary fig. A5, Supplementary Material online). Among the *aexU*-positive genomes, the major clade contained strains from the Hydrophila phylogroup, a second clade contained strains from the Veronii phylogroup, and a third contained *A. encheleia* and *A. piscicola* strains.

Finally, T3SS component and effector phylogenies showed major discrepancies between them and phylogenomics, which suggested HGT events. The frequency, distribution and location of genes encoding T3SS components and effectors do not support the case for unique ancestral inheritance. The T3SS-related genes were more than likely acquired by HGT events that involved only some ancestors among phylogroups. The nucleotide Blast analysis demonstrated the relatedness of aeromonad genetic sequences to *Pseudomonas* sp. strains for genes encoding T3SS components and *aexT* and to *Chromobacterium* sp. strains for *aexU* (data not shown).

For other toxins, the gene *ast* was detected in 19% of strains, exclusively in the Hydrophila, Salmonicida, and Veronii phylogroups (supplementary fig. A6, Supplementary Material online). The ML tree of *ast* sequences showed one major clade that corresponded to the group Hydrophila. The other *ast*-positive genomes were more scattered and found close in the Salmonicida group (*A. piscicola* and *A. bestiarum*) or separated from each other, as in the Veronii group (*A. sobria* and *A. enteropelogenes*). This distribution suggested HGT acquisition of *ast* in a Hydrophila ancestor and in some isolated lineages. The nucleotide Blast analysis demonstrated the similarity of aeromonad genetic sequences with *Enterobacteriaceae* strains for *ast* (data not shown).

CDSs homologous to the toxin RtxA were found in 14% of aeromonads, mainly in the Hydrophila phylogroup. This CDS was associated with a region upstream of an RTX toxin that activates lysine acyltransferase. The genetic clustering and the locus organizations of the *rtxA* gene were different among the two taxonomic species of the Hydrophila phylogroup, which argues for an acquisition by two lateral transfer events, perhaps from vibrios given the relatedness of *rtx* sequences seen in Blast analysis (data not shown), one by a common ancestor of the members of *A. hydrophila* and another to the members of *A. dhakensis* (supplementary fig. A7, Supplementary Material online).

Nine aeromonad genomes (i.e., 14%) contained a gene coding for a putative exotoxin A that was very closely related to one found in *P. aeruginosa* (70% identity from 89% of the total sequence length in more than 100 different strains). This CDS was found almost exclusively in the strains within the Hydrophila phylogroup, with a conserved gene sequence and similar genetic organization (supplementary fig. A8, Supplementary Material online, and table 2).

Finally, contrary to the thermolabile cytotonic enterotoxin (Alt) gene that nearly met the threshold for inclusion in the core genome (97%), the genes encoding ADP-ribosylating toxins (AexT, AexU), RtxA toxin, exotoxin A, and thermostable cytotonic enterotoxin (Ast) were quite rare within the studied genomes (11–20%). When present, a low level of divergence was observed between strains from the same phylogroup (supplementary figs. A4–A8, Supplementary Material online). These features for *aexT*, *aexU*, *exoA*, and *ast* support the presumption of horizontal acquisition via MGEs and with a higher exchange compatibility among closely related strains.

## Discussion

The traditional approach to characterize bacterial pathogenesis is mutagenesis/complementation and assessment of phenotypes in model systems. Although they are generally an efficient approach for specialist pathogens, these methods can lead to equivocal results for opportunistic pathogens such as *Aeromonas* (e.g., Gavín et al. 2002; Sha et al. 2002; Vilches et al. 2008; Sierra et al. 2010; Ponnusamy et al. 2016). Furthermore, these methods have largely failed to attribute virulence-associated genes in *Aeromonas* in accordance with molecular Koch’s postulates (Falkow 2004). Population studies that have compared the pathogenicity of environmental strains with that of clinical/animal strains using standard molecular biology techniques have delivered disappointing results in part because the high genetic diversity of the so-called virulence-associated genes in these bacteria has rendered polymerase chain reaction-based tools unreliable (Talagrand-Reboul et al. 2018). To better understand the adaptation mechanisms that drive the genome dynamics in these environmental opportunistic bacteria, in this study, we compared the phylogenomics of several virulence genes against the genetic background of the genus *Aeromonas*. We selected 11 previously described virulence factors with characterized phenotypes for this analysis, that is, T3SS and effector proteins, flagellar protein, collagenase, serine protease, cytotoxic, and cytotonic enterotoxins (Bernheimer et al. 1975; Chopra et al. 1996; Abrami et al. 1998; Braun et al. 2002; Gavín et al. 2002; Sha et al. 2002, 2007; Vilches et al. 2004; Suarez et al. 2012; Duarte et al. 2015; Ponnusamy et al. 2016). We tested the hypothesis that the evolutionary process of virulence-associated genes is informative of pathoadaptation. The method used to study the synteny of genes takes slight rearrangements into consideration to formulate evolution hypotheses. Our analysis revealed a great complexity with diverse evolution modes and gene organization and an uneven distribution of virulence genes in the genus, with evidence of HGT among strains in some cases. Overall, this study provided insights into aeromonad pathoadaptation or the ability of some members of this group to emerge as pathogens.

### Pathogenicity versus Pathogenomics

The 30 species within the genus *Aeromonas* are not equivalent in their clinical importance. Human infections are most often caused by four taxonomic species that represent 96% of the aeromonads found in clinical samples: *A. caviae* (30%), *A. dhakensis* (26%), *A. veronii* (22%), and *A. hydrophila* (18%) (Figueras and Beaz-Hidalgo 2015). The species *A. schubertii*, *A. enteropelogenes*, *A. jandaei*, *A. allosaccharophila*, *A. encheleia*, *A. sanarellii*, and *A. taiwanensis* are rarely involved in human aeromonosis (Lai et al. 2007; Alperi et al. 2010; Roger et al. 2012; Latif-Eugenín et al. 2016; Sinclair et al. 2016). The pathogenicity profiles available for the species *A. media*, *A. rivipollensis*, *A. eucrenophila*, *A. encheleia*, and *A. tecta* are uncertain (Demarta et al. 2008; Roger et al. 2012; Talagrand-Reboul et al. 2017), whereas other species (e.g., *A. salmonicida*, *A. popoffii*, and *A. bestiarum*) have rarely been isolated from clinical samples (Hua et al. 2004; Yang et al. 2008; Sinclair et al. 2016). From our analysis, virulence-associated gene distribution could help to explain the pathogenic potential of *A. hydrophila* and *A. dhakensis* because these strains presumably contained most of the target virulence genes, but this hypothesis suffers from a possible bias related to the nature of the gene panel.

A recent study examined the pathogenicity and extensive virulence arsenal of *A. dhakensis* (Chen et al. 2016), and the data correlated well with the pathogenomic profile described in our work. Additional genome analyses suggest a greater virulence potential in *A. hydrophila* in comparison to *A. veronii* and *A. caviae* (Ghatak et al. 2016). Certain diseases/conditions have been associated with a particular aeromonad; however, our understanding of this relationship is limited. For instance, *A. caviae* and *A. veronii* are highly prevalent in enteritis and bacteremia, whereas *A. hydrophila* and *A. veronii* are prevalent in wound infections (Lamy et al. 2009). These observations suggest different capabilities in invasion and tissue damage between phylogroups. In our study, fewer virulence genes were identified in *A. veronii* and *A. caviae* strains compared with *A. hydrophila*. Most notably, aerolysin and T3SS components or effectors were absent from all *A. caviae* strains. Interestingly, the observed virulence profile of *A. caviae* was similar to that of *A. media*, but *A. caviae* exhibited higher infectious success (Lamy et al. 2009; Figueras and Beaz-Hidalgo 2015). We must also consider that *A. caviae* or *A. veronii* strains may possess unknown virulence determinants not included in our study and that the selected strains may not be representative enough of the virulence gene prevalence in these species. An unbiased pangenome survey could strengthen targeted studies and lead to a more robust correlation between pathogenomics and pathogenesis in aeromonads, as demonstrated for *P. aeruginosa* with the aim of searching for new drugs and vaccines (Mosquera-Rendón et al. 2016) or recently for the identification and characterization of new putative *Aeromonas* spp. T3SS effectors (Rangel et al. 2019). One limitation of this study, as in any genomic survey without phenotypic and/or proteomic investigation, is that virulence factor gene detection does not ensure appropriate protein expression or efficient secretion, where applicable. Despite these limitations, our work provides novel results that contribute to our understanding of virulence in aeromonads.

### Virulence by Exaptation

From our analysis, neither the isolation source nor the degree of reported pathogenicity influenced the results of the virulence screen. The presence of virulence genes in environmental aeromonads is not rare and was also observed by Vázquez-Rosas-Landa et al. (2017) using a similar genomic approach. In that study, the authors reported that the natural selection of these so-called “virulence genes” in aquatic bacteria reflects the integral nature of these factors to the lifestyle within that particular habitat. For instance, secretion systems or motility mechanisms may enhance fitness by facilitating nonpathogenic interactions with other bacteria and eukaryotes (Silver et al. 2007). Thus, adaptive factors that are primarily of ecological importance within one niche are thought to become virulence factors by exaptation, enabling aeromonads to emerge as pathogens and cause disease in incidental hosts.

The analysis of Alt and ColAh may illustrate the hypothesis of exaptation-based virulence in aeromonads. ColAh is a peptidase that belongs to the gluzincin subfamily of the M9 family recently described in *A. piscicola* that shares low similarity with other known bacterial collagenases. The enzyme exhibits a cytopathic effect on Vero cells (Duarte et al. 2015), but its overall role in aeromonad pathogenicity is mostly unknown. ColAh may play a role in host invasion similar to other bacterial collagenases. The cytotonic enterotoxin Alt belongs to the enterotoxic arsenal of aeromonads. However, Alt contributed to *A. hydrophila*-induced gastroenteritis in a mouse model to a lesser extent than the toxin Act (Sha et al. 2002). The presence of the *alt* gene in the Aeromonas “soft-core genome” (≥95% of genomes) (Kaas et al. 2012), including exclusively environmental nonpathogenic species (e.g., *A. rivuli*), raises the question about its true involvement in pathogenicity. In the literature, the prevalence of *alt* is relatively low for a gene that we can consider as belonging to the soft-core genome (e.g., 53% of 129 strains, Aravena-Román et al. 2014). One reason for this is that polymerase chain reaction assays can have poor sensitivity at the genus level because of the high polymorphism that results in a biased *alt* prevalence (Talagrand-Reboul et al. 2018). From our analysis, the genes *alt* and *colAh* were highly conserved in the genus *Aeromonas* because they were found in the large majority of the genomes (i.e., 97% and 89%, respectively), and their phylogenies were congruent with the global genetic background of the whole genus. We assume that these genes may have been acquired by a common ancestor and then followed the general genetic evolution of these bacteria. With their basic functions as lipases and proteases, Alt and ColAh are likely involved in general aeromonad metabolism within the customary environment. In a secondary host environment, these enzymes can be utilized in other biological processes, such as those involved in virulence. These secondary functions represent potential roles in exaptation (Adiba et al. 2010; Cabello and Godfrey 2018) for these two enzymes.

### Aer Is Likely a True Virulence Factor

Aerolysin (Aer) is a pore-forming toxin secreted by *Aeromonas* (Bernheimer and Avigad 1974; Bücker et al. 2011). Aer/Act is considered the major enterotoxin that contributes to aeromonad pathogenicity (Sha et al. 2002), and as such, its presence/absence in a genome should be related to the pathogenic behavior of the strain. Our observations are consistent with this hypothesis. First, *act*/*aer*-negative species exhibit little to no virulence. *Aeromonas media* displays a rather low virulence profile in humans and animals (Talagrand-Reboul et al. 2017). To date, *A. fluvialis* has not been reported as virulent in any host, and *A. simiae* has only been described in monkey feces but not associated with any pathology (Harf-Monteil et al. 2004). Second, among the recognized pathogenic aeromonads, the Aer/Act-negative species *A. caviae* is less cytotoxic than the Aer/Act-positive species *A. veronii* (Chen et al. 2015). Finally, for almost all studied isolates of *A. dhakensis*, a species very closely related to *A. hydrophila* that is one of the most virulent among aeromonads (Chen et al. 2016), a specific *act*/*aer* gene was found. The theory that Aer/Act produced by *A. dhakensis* may contribute to its high virulence potential requires further investigation.

We observed a significant genetic association between the *aer*/*act* and *ser* genes that is probably related to their function (Iacovache et al. 2016). The *aer*+/*ser*− pattern suggests that either proteases other than Ser could be involved in the activation of the pore-forming aerolysin or Aer/Act is secreted but not matured in the transmembrane complexes of these strains. Despite their association, the phylogenies of *aer*/*act* and *ser* are complex and distinct. The phylogeny reconstructed from *aer*/*act* leads us to assume that two different *Aeromonas* ancestors acquired variants of the aerolysin genes, and this gene may be associated with the speciation process. In contrast, it seems likely that the *ser* gene was acquired by a common ancestor of aeromonads and then transmitted by vertical inheritance. It could have been positively coselected in the *aer*/*act*-positive *Aeromonas* clades, but the pleiotropic role of proteases may explain its presence in several *aer*/*act*-negative genomes.

### LafA, an Evolution Mode toward Multiple Copies

Lateral flagellin is the major component of lateral flagella that are involved in swarming motility and biofilm formation in numerous bacteria. Mesophilic *Aeromonas* strains display a polar flagellum but can express multiple lateral flagella (Kirov et al. 2002). Mutagenesis and complementation experiments confirmed that the lateral flagella of *Aeromonas* play a role in adherence and biofilm formation (Gavín et al. 2002). Despite numerous orthologs, the lateral flagellar system in *Aeromonas* does not share either structural or regulatory genes with the polar flagellar system (Wilhelms et al. 2013). In this work, we hypothesized that after acquisition from a common ancestor, duplication likely drove the evolution of the lafA gene in the genus *Aeromonas* and that copies of the gene were also involved in HGT events. This confirms the major role of the multiple *lafA* copies in the genetic evolution of bacterial flagella and agrees with previous studies of Proteobacteria in that phylogenetic analysis and organization of lateral flagellar genes highly suggest that this system originated both from the duplication and horizontal transfer of polar flagella system genes (Liu and Ochman 2007). From a functional view, the four different copies detected in the *A. diversa* genome are possibly involved in the peculiar swarming ability of the species (Miñana-Galbis et al. 2010), although the swarming capacity has not been studied with the same approach in other members of the genus.

### T3SS, an Example of Pathoadaptative Evolution

T3SSs enable the injection of effectors into eukaryotic cells. They are widely distributed in Gram-negative bacteria. The T3SS found in aeromonads is described as a homolog of those reported in *P. aeruginosa* and *Yersinia* spp., suggesting its potential role in *Aeromonas* pathogenesis with the ADP-ribosylating toxins AexT and AexU as translocons, as supported by mutagenesis data (Burr et al. 2002; Vilches et al. 2004; Sierra et al. 2010). The *aexU* null mutant was attenuated in a mouse model (Sierra et al. 2010). In *A. hydrophila*, the *aexT* mutant showed a slight reduction in virulence, whereas mutants without a functional T3SS apparatus displayed significantly reduced virulence in the same assays (Vilches et al. 2008). In addition to causing cellular damage, the T3SS and related effectors in *A. salmonicida* impair the transcription of immune mediators in rainbow trout (Origgi et al. 2017).

From our phylogenetic analysis and study of the distribution/organization of the loci, we hypothesize that aeromonad T3SS-related genes (T3SS and effectors) were acquired by HGT within phylogroups, which is consistent with another work that investigated the distribution and genetic evolution of 21 *Aeromonas* T3SS likely effector families from 105 strains covering the whole genus (Rangel et al. 2019) and observed in other genera, for example, *Vibrio* (Okada et al. 2010) and *Pseudomonas* (Dillon et al. 2019). The T3SS effectors AexT and AexU likely correspond to two different allelic forms of ADP-ribosyltransferase, similar to the HopZ gene family, which encodes T3SS effectors in the opportunistic plant pathogen *Pseudomonas syringae*. Briefly, T3SS effectors corresponded to three allelic forms of HopZ, HopZ1, HopZ2, and HopZ3, which display various specific targets or substrates. HopZ proteins are structurally and functionally heterogeneous due to 1) the acquisition of HopZ2 and HopZ3 from members of the genera *Xanthomonas* and *Erwinia* by HGT, respectively and 2) the pathoadaptive evolution of the ancestral form HopZ1. The evolution of hopZ1 follows the evolution of the genes from the *P. syringae* core genome (Ma et al. 2006). Interestingly, previous works on the genetics of T3SS effectors of *P. syringae* have shown that pathoadaptation is not inconsistent with genomic plasticity or the acquisition of virulence genes by lateral transfer, but T3SS effectors can be affected by mutations, which can then modify the functions of the effectors (Ma et al. 2006; Dillon et al. 2019).

In this work, the examination of the *aexU*-flanking genes by RAST annotation showed a possible association between *aexT* and *aexU* in *A. veronii* strains, as previously reported (Silver and Graf 2009). We observed that the presence of *aexU* in addition to *aexT* was an original feature of *A. veronii* strains among the genus *Aeromonas*, but this association was not present in all the strains of the species. Our results were highly consistent with those reported in the recent study of Rangel et al. (2019) on aeromonad T3SS effectors.

Finally, a functional study demonstrated the regulatory crosstalk between the T3SS and lateral flagellum systems (Zhao and Shaw 2016), but no obvious evolutionary link between *lafA* and the T3SS genes was observed therein (figs. 4 and 5).

In summary, genes encoding the T3SS structural components and the effectors AexT and AexU in the genus *Aeromonas* have probably followed a pathoadaptive evolution likely guided by their environment and/or their host. The acquisition of the genes and their subsequent evolution may have been driven by interactions with eukaryotic organisms within their native aquatic environments (e.g., amoeba, nematodes, or leech) and by inadvertently encountered circumstances where the T3SS and their effectors act as virulence factors (Yu et al. 2004; Sha et al. 2007). This hypothesis requires further study to discern the selective pressures leading to maintenance, expression, and induction of the T3SS machinery.

### HGT as a Major Driver for Toxin Acquisition

Two mechanisms of HGT have been described in *Aeromonas* bacteria, that is, transformation and conjugative transfer (Piotrowska and Popowska 2015). The interconnected network generated by the neighbor-net analysis, the high number of ISs detected, and the presence of putative ICEs and IMEs are consistent with the probable frequency of these HGT events among aeromonads. Conversely, the analysis of IMEs and ICEs did not show any significant impact of particular conjugative events on the presence of the studied virulence factor genes. Despite the absence of direct imputation of well-identified transfers, the comparative analysis results of the genus phylogeny and the studied genes are still compatible with HGT. These transfers may have occurred, independently of conjugal events, by DNA transformation mechanisms, which have been experimentally demonstrated in *Aeromonas*, and facilitated exchanges were more frequent between closely related strains (Huddleston et al. 2013).

Cumulative data on the three non-T3SS-related toxins that we evaluated (*ast*, *rtxA*, and *exoA*) showed that they were likely acquired by HGT from other environmental/aquatic bacteria, such as *Pseudomonadaceae*, *Enterobacteriaceae*, or vibrios. These toxins are particularly associated with the Hydrophila phylogroup, *A. hydrophila* species for the gene *ast*, *A. dhakensis* species for *exoA*, and both species for *rtxA*. The *exoA* gene detected in *A. hydrophila* genomes codes for a homolog of *P. aeruginosa* exotoxin A, a major virulence factor for this bacterium. Mutagenesis experiments showed that *exoA* is associated with host tissue destruction, which allows invasive deep infections, such as necrotizing fasciitis, in a murine model (Ponnusamy et al. 2016). We found that this gene was particularly frequent in the highly virulent species *A. dhakensis* (6/6) (Chen et al. 2016). We hypothesize that these three toxins, for which a high degree of virulence in animal models has been demonstrated, contribute to a particular pathogenicity of members of the phylogroup Hydrophila. Moreover, these toxins may exhibit their virulence properties when produced alone and/or in conjunction or sequentially with other toxins, potentially resulting in aggregate adverse effects.

### Aeromonads and Pathoadaptation

The substantial diversity of evolutionary modes for virulence-associated genes can give rise to a complex evolutionary network and likely sets the foundation for different assemblages of virulence factors in aeromonads. Most of the virulence-associated genes are chromosomal, which implies that either their functions are fundamentally essential or beneficial or that they have been fixed after HGT as determinants involved in niche adaptation. The dynamics of adaptive evolution through changes in gene sequence, regulation, expression, loss, or acquisition correspond to the pathoadaption phenomenon presumably enhance the fitness of a microbe in its new host niche (Pallen and Wren 2007).

In *Aeromonas*, the products of the *alt*, *colAh*, and, to some extent, *ser* genes are thought to serve functions essential to aeromonad biology and physiology because these genes follow vertical evolution patterns parallel to the evolution patterns of genes of the core genome. These hydrolytic enzymes are probably involved in general cell metabolism, but by exaptation (Adiba et al. 2010; Cabello and Godfrey 2018), they may also be involved in interactions with the host and virulence. HGT is another mode of evolution detected herein for more specialized virulence-associated genes, such as toxins RtxA, ExoA, Ast, AexT, and AexU. Some of these virulence-associated genes were specific to a species or phylogroup. However, none of them was the sole virulence factor in a pathogenic phenotype. Some of these genes were also probably lost by several strains, for example, aerolysin in *A. hydrophila*. Another signature of gene loss in aeromonads is the interruption *of* several virulence-associated genes by ISs. The accumulation of pseudogenes and insertion elements for *A. salmonicida* strains corresponds to genomic markers of the shift to a new niche (Reith et al. 2008; Aujoulat et al. 2012; Vincent et al. 2016). Indeed, the psychrophilic subspecies of *A. salmonicida* are considered specialized pathogens with a narrow host range limited to fish (Vincent et al. 2016). In addition, recent works have highlighted the degree of diversity of virulence traits among mesophilic *A. salmonicida* strains (Vincent et al. 2019), and these results may indicate that further studies of virulence gene evolution are needed to increase the understanding of pathoadaptation in *A. salmonicida* lineages.

## Conclusion

In conclusion, virulence-associated gene content by itself does not fully explain the pathogenic behavior of *Aeromonas* taxonomic species either in vitro or in clinical contexts. However, the high complexity of *Aeromonas* virulence genes in terms of uneven distribution, diversity of organization, and variable evolutionary modes revealed in this study likely explains why *Aeromonas* pathogenicity is so difficult to assess in terms of attribution. The pathogenomics overview in this study postulates that *Aeromonas* virulence-associated genes should be studied at the population level and that studies performed on type or model strains in a species should not be generalized to the whole species. In addition, correlations between pathogenomics and epidemiological data should also be considered. In a recent study, the analysis of WGS for 101 *A. salmonicida* subsp. s*almonicida* strains has revealed four major lineages of this fish pathogen that emerged in Denmark, and the genomic variations of these strains were associated with virulence factors carried on and disseminated by plasmids (Bartkova et al. 2017). We emphasize that pathoadaptation resulting in diverse phenotypic variations is a presumptive key element to consider in the challenging effort to determine virulence profiles and discrete pathotypes in the *Aeromonas* genus.

Finally, the present study provides a novel point of view of evolutionary processes concerning virulence genes in a model of environmental opportunistic pathogen bacteria. We anticipate that the dichotomies of vertical/lateral inheritance, prevalence in the genus/lineages, and single/multiple copies of the virulence genes will be established in future genomic analyses to characterize newly described virulence factors. Our pathogenomic analysis allows a better understanding of the dynamics of the emergence and evolution of pathogenic traits in aeromonads. The varied patterns of evolution suggested by our study, exaptation processes, fixation in the chromosome of virulence factors acquired by HGT, virulence-associated genes that evolve according to core genome phylogeny, and loss of virulence genes in specific niches are converging characteristics consistent with the role of niche adaptation, including pathoadaptation, in aeromonad speciation. This vision of aeromonads evolution meets current evolution theories such as the extended evolutionary synthesis that is influenced by various disciplines such as ecology. This synthesis suggests that organisms do not evolve to fit into preexisting environments but coconstruct and coevolve with their environments, thereby considered as ecological niches (Laland et al. 2014). For a pathogen, changing the structure of its ecosystem involves coevolution with the host and the construction of a niche that allows contact with this host.

## Supplementary Material

evaa055_Supplementary_Data

## Notes

**Data deposition:** New bacterial WGS released from this project have been deposited at NCBI Genome under the accession numbers JAAALX000000000, JAAALW000000000, JAAALV000000000, and JAAALU000000000.
