# Supplementary material for: Comparative and Evolutionary Genomics of Isolates Provide Insight into the Pathoadaptation of Aeromonas
Source: Genome Biol Evol. 2020 Mar 20;12(5):535–52. doi: 10.1093/gbe/evaa055 (PMC7250499; doi:10.1093/gbe/evaa055)
Supplement: evaa055_Supplementary_Data [file evaa055_supplementary_data.zip › Supp/Supplemental Figures revised MS 280120.docx]

**Appendix A. Supplementary material**

**Supplementary Fig. A1. Neighbour-network graph based on the aligned SNP sequences (27,856 SNPs) identified in whole genome sequences of the 64 *Aeromonas* spp.**

**Supplementary Fig. A2. Maximum-Likelihood tree based on *colAh* gene sequences (2,772 nt.)** reconstructed using GTR model plus gamma distribution as a substitution model with 55 complete nucleotide sequences. The two interrupted sequences (*A. caviae* FDAARGOS 72 and *A. salmonicida* subsp. *smithia* homologs of *colAh*) are not represented in this tree. The horizontal lines represent genetic distance, with the scale bar indicating the number of substitutions per nucleotide position. The numbers at the nodes are support values estimated with 100 bootstrap replicates. Only bootstrap values ≥70 are indicated. The major phylogenetic group of each strain is indicated by coloured ranges on strain labels. The type of genetic organization of the flanking genes shown in the inserted box is indicated for each strain with a numbered star.

**Supplementary Fig. A3. Maximum-Likelihood tree based on** ***ser* gene sequences (1,911 nt.)** reconstructed using the TN model plus gamma distribution as a substitution model from 41 complete *ser* nucleotide sequences. The three interrupted sequences (*A. rivipollensis* BVH40, *A. hydrophila* BVH25a and *A. salmonicida* subsp. *smithia* homologs of *ser*) are not represented in this tree. The numbers at the nodes are support values estimated with 100 bootstrap replicates. Only bootstrap values ≥70 are indicated. The major phylogenetic group of each strain is indicated by coloured ranges on strain labels. The type of genetic organization of the flanking genes shown in the inserted box is indicated for each strain with a numbered star.

**Supplementary Fig. A4. Maximum-Likelihood tree based on** ***aexT* gene sequences (1,434 nt.)** reconstructed using the TIM2 model as a substitution model from 8 complete *aexT* nucleotide sequences. The numbers at the nodes are support values estimated with 100 bootstrap replicates. Only bootstrap values ≥70 are indicated. The major phylogenetic group of each strain is indicated by coloured ranges on strain labels. The type of genetic organization of the flanking genes shown in the inserted box is indicated for each strain with a numbered star.

**Supplementary Fig. A5. Maximum-Likelihood tree based on *aexU* gene sequences (1,554 nt.)** reconstructed using the TIM model plus gamma distribution as a substitution model from 13 complete *aexT* nucleotide sequences. The numbers at the nodes are support values estimated with 100 bootstrap replicates. Only bootstrap values ≥70 are indicated. The major phylogenetic group of each strain is indicated by coloured ranges on strain labels. The type of genetic organization of the flanking genes shown in the inserted box is indicated for each strain with a numbered star.

**Supplementary Fig. A6.** **Maximum-Likelihood tree based on** ***ast* gene sequences (1,920 nt.)** reconstructed using the TVM model plus gamma distribution as a substitution model from 11 complete *aexT* nucleotide sequences. One interrupted sequence (*A. hydrophila* BVH 25a homolog of *ast*) was not represented in this tree. The numbers at the nodes are support values estimated with 100 bootstrap replicates. Only bootstrap values ≥70 are indicated. The major phylogenetic group of each strain is indicated by coloured ranges on strain labels. The type of genetic organization of the flanking genes shown in the inserted box is indicated for each strain with a numbered star.

**Supplementary Fig. A7.** **Maximum-Likelihood tree based on** ***rtxA* gene sequences (16,118 nt.)** reconstructed using a GTR model plus gamma distribution as a substitution model from 8 complete *rtxA* nucleotide sequences. One interrupted sequence (*A. salmonicida* subsp. *pectinolytica* homolog of *rtxA*) was not represented in this tree. The numbers at the nodes are support values estimated with 100 bootstrap replicates. Only bootstrap values ≥70 are indicated. The major phylogenetic group of each strain is indicated by coloured ranges on strain labels. The type of genetic organization of the flanking genes shown in the inserted box is indicated for each strain with a numbered star.

**Supplementary Fig. A8. Maximum-Likelihood tree based on** ***exoA* gene sequences** **(1,935 nt.)** reconstructed using the TVM model plus gamma distribution as a substitution model from 8 complete *rtxA* nucleotide sequences. One interrupted sequence (*A. salmonicida* subsp. *pectinolytica* homolog of *exoA*) was not represented in this tree. The numbers at the nodes are support values estimated with 100 bootstrap replicates. The major phylogenetic group of each strain is indicated by coloured ranges on strain labels. The type of genetic organization of the flanking genes is shown in the inserted box.
