# Supplementary material for: Comparative and Evolutionary Genomics of Isolates Provide Insight into the Pathoadaptation of Aeromonas
Source: Genome Biol Evol. 2020 Mar 20;12(5):535–52. doi: 10.1093/gbe/evaa055 (PMC7250499; doi:10.1093/gbe/evaa055)
Supplement: evaa055_Supplementary_Data [file evaa055_supplementary_data.zip › Supp/Supplemental Tables revised version 280120.docx]

**TABLE S1. Description of the studied *Aeromonas* strains and genomes**

| *Aeromonas* species | Strain | Source of strain isolation | Genome size (Mbp) | No of scaffolds | Average genome coverage | N_50_f (nt) | G+C content (%) | No of predicted CDSs | No of predicted ISs^c^ | No of predicted ICEs/IMEs^d^  (length in bp) | Virulence associated- genes of the panel located in putative ICEs/IMEs | Quality of genome assembly | Accession number |
| --- | --- | --- | --- | --- | --- | --- | --- | --- | --- | --- | --- | --- | --- |
| *A. allosacharophila* | CECT 4199^T^ | Infected eel | 4.66 | 120 | 87 | 114,541 | 58.4 | 4,186 | 41 | 2 IMEs (86,550 bp and 52,559 bp) | - | IHQ | PRJEB7019^a^ |
| *A. aquatica* | CECT 8025^T^ | Water | 4.58 | 171 | 25 | 67,531 | 61.2 | 4,097 | 6 | 0 | - | HQ | JRGL01^b^ |
| *A. australiensis* | CECT 8023^T^ | Irrigation water system | 4.11 | 113 | 128 | 95,095 | 58.1 | 3,733 | 13 | 0 | - | IHQ | PRJEB7021^a^ |
| *A. bestiarum* | CECT 4227^T^ | Fish | 4.68 | 41 | 53 | 237,067 | 60.5 | 4,223 | 6 | 0 | - | IHQ | PRJEB7022^a^ |
| *A. bivalvium* | CECT 7113^T^ | Cockles | 4.28 | 69 | 30 | 149,050 | 62.3 | 3,909 | 13 | 1 IME w/o identified DR (6,053 bp) | - | IHQ | PRJEB7023^a^ |
| *A. caviae* | CECT 838^T^ | Guinea pig | 4.47 | 111 | 95 | 101,663 | 61.6 | 4,081 | 7 | 1 IME (5,5374bp) | - | IHQ | PRJEB7024^a^ |
| *A. caviae* | CECT 4221 | Used oil emulsions | 4.58 | 332 | 66 | 31,465 | 61.0 | 4,207 | 26 | 0 | - | IHQ | PRJEB7046^a^ |
| *A. caviae* | ADV118 | Human intra-abominal hematoma | 4.39 | 140 | 93 | 61,541 | 61.5 | 3,982 | 15 | 0 | - | IHQ | JAAALX000000000^a^ |
| *A. caviae* | BVH84 | Human stool | 4.48 | 151 | 77 | 80,861 | 61.3 | 4,074 | 19 | 0 | - | IHQ | JAAALW000000000^a^ |
| *A. caviae* | BVH98 | Human wound | 4.46 | 159 | 121 | 69,804 | 61.4 | 4,070 | 27 | 1 IME w/o identified DR (8,838 bp) | - | IHQ | JAAALV000000000^a^ |
| *A. caviae* | AK245 | Lake water | 4.45 | 203 | 92 | 50,267 | 61.6 | 4,081 | 22 | 1 IME (11,598 bp) | - | IHQ | JAAALU000000000^a^ |
| *A. caviae* | FDA ARGOS 72 | Human diarrheic stool | 4.52 | 1 | 22 | 4,517,515 | 61.7 | 4,397 | 14 | 1 IME (13,353 bp) | - | HQ | JTBK01^b^ |
| *A. dhakensis* | CECT 7289^T^ | Aquaria of ornamental fish | 4.69 | 78 | 117 | 163,504 | 61.7 | 4,266 | 2 | 0 | - | IHQ | PRJEB7020^a^ |
| *A. dhakensis* | CIP 107500 | Human diarrheic stool | 4.71 | 73 | 84 | 165,885 | 61.8 | 4,284 | 6 | 0 | - | IHQ | PRJEB7048^a^ |
| *A. dhakensis* | BVH28b | Human wound | 4.89 | 68 | 130 | 150,860 | 61.7 | 4,466 | 10 | 0 | - | IHQ | PRJEB9016^a^ |
| *A. dhakensis* | SSU | Human diarrheic stool | 4.94 | 2 | 285 | 4,791,870 | 61.5 | 4,449 | 8 | 2 IMEs (18,220 bp and 14,014 bp)  1 ICE (68,526 bp) | - | HQ | AGWR01^b^ |
| *A. dhakensis* | AAK1 | Human blood | 4.76 | 37 | 21 | 404,457 | 61.8 | 4,290 | 15 | 1 IME (41,862 bp) | - | HQ | BAFL01^b^ |
| *A. dhakensis* | F2S2-1 | Fish skin | 4.75 | 64 | 1 | 260,482 | 61.8 | 4,244 | 5 | 0 | - | SD | LZFM01^b^ |
| *A. diversa* | CECT 4254^T^ | Human wound | 4.06 | 37 | 116 | 203,531 | 61.5 | 3,711 | 12 | 0 | - | IHQ | PRJEB7026^a^ |
| *A. encheleia* | CECT 4342^T^ | Fish | 4.47 | 35 | 112 | 380,984 | 61.9 | 4,076 | 4 | 0 | - | IHQ | PRJEB7027^a^ |
| *A. enteropelogenes* | CECT 4255^T^ | Human stool | 4.34 | 27 | 66 | 640,249 | 60.0 | 3,917 | 5 | 1 IME (8,833 bp) | - | IHQ | PRJEB7043^a^ |
| *A. eucrenophila* | CECT 4224^T^ | Fresh water fish | 4.54 | 22 | 50 | 441,212 | 61.1 | 4,113 | 2 | 0 | - | IHQ | PRJEB7029^a^ |
| *A. finlandiensis* | CECT 8028^T^ | Lake water | 4.72 | 376 | 16 | 31,035 | 58.6 | 4,207 | 11 | 0 | - | SD | JRGK01^b^ |
| *A. fluvialis* | LMG 24681^T^ | River water | 3.90 | 76 | 48 | 108,949 | 58.2 | 3,609 | 26 | 1 ICE (26,120 bp) | - | IHQ | PRJEB7030^a^ |
| *A. hydrophila* subsp. *hydrophila* | CECT 839^T^ | Tin of milk with fishy odor | 4.74 | 1 | NA | 4,744,448 | 61.5 | 4,119 | 3 | 0 | - | complete | CP000462^b^ |
| *A. hydrophila* | BVH25a | Human respiratory tract | 5.10 | 130 | 44 | 84,371 | 60.9 | 4,598 | 19 | 1 ICE (69,288 bp) | - | IHQ | PRJEB9013^a^ |
| *A. hydrophila* subsp. *ranae* | CIP 107985 | Septicemic frog | 4.68 | 107 | 140 | 90,304 | 61.6 | 4,268 | 5 | 1 IME (29,916 bp) | - | IHQ | PRJEB7049^a^ |
| *A. hydrophila* | AH10 | Fish | 4.91 | 1 | NA | 4,908,265 | 61.1 | 4,391 | 13 | 1 IME (20,229 bp) | - | complete | CP011100^b^ |
| *A.hydrophila* | AHNIH1 | Human rectum | 5.05 | 2 | NA | 4,906,118 | 61.0 | 4,491 | 68 | 1 IME (36,792 bp) | - | complete | PRJNA273636^b^ |
| *A. hydrophila* | AL06-06 | Infected fish | 4.90 | 4 | NA | 4,901,389 | 61.4 | 4,414 | 19 | 0 | - | complete | PRJNA270887 |
| *A. hydrophila* | ML09-119 | Infected fish | 5,02 | 1 | NA | 5,024,500 | 60.8 | 4,472 | 18 | 0 | - | complete | CP005966^b^ |
| *A. jandaei* | CECT 4228^T^ | Human stool | 4.50 | 58 | 55 | 161,393 | 58.7 | 4,065 | 3 | 1 IME w/o identified DR (30,956 bp) | - | IHQ | PRJEB7031^a^ |
| *A. lacus* | CECT 8024^T^ | Water | 4.39 | 196 | 29 | 79,727 | 59.0 | 3,914 | 11 | 1 ICE (424,626 bp) | - | HQ | JRGM01^b^ |
| *A. media* | CECT 4232^T^ | River water | 4.48 | 233 | 60 | 37,608 | 61.1 | 4,075 | 28 | 1 IME w/o identified DR (4,464 bp) | - | IHQ | PRJEB7032^a^ |
| *A. media* | LMG 13464 | Infected fish | 4.45 | 99 | 87 | 103,746 | 61.3 | 4,014 | 21 | 0 | - | IHQ | PRJEB12347^a^ |
| *A. media* | CECT 7111 | Oyster | 4.41 | 92 | 70 | 108,504 | 61.6 | 3,998 | 8 | 0 | - | IHQ | PRJEB12345^a^ |
| *A. molluscorum* | CIP 108876^T^ | Wedge-shells | 4.23 | 309 | 9 | 21.565 | 59.2 | 3,946 | 14 | 1 IME (17,627 bp) | - | SD | AQGQ01^b^ |
| *Aeromonas sp.* genomosp. *paramedia* | CECT 8838 | Human diarrheic stool | 4.46 | 128 | 99 | 78,349 | 62.2 | 4,086 | 8 | 0 | - | IHQ | PRJEB12349^a^ |
| *A. piscicola* | LMG 24783^T^ | Salmon | 5.18 | 91 | 99 | 150,424 | 59.0 | 4,713 | 24 | 0 | - | IHQ | PRJEB7033^a^ |
| *A. popoffii* | CIP 105493^T^ | Drinking water production plant | 4.76 | 105 | 67 | 113,495 | 58.4 | 4,331 | 31 | 1 IME (13,550 bp) | - | IHQ | PRJEB7034^a^ |
| *A. rivipollensis* | LMG 13459^T^ | Infected fish | 4.49 | 111 | 76 | 107,760 | 61.7 | 4,091 | 26 | 0 | - | IHQ | PRJEB12346^a^ |
| *A. rivipollensis* | 76c | Human diarrheic stool | 4.69 | 137 | 79 | 93,768 | 61.3 | 4,255 | 33 | 1 IME (38,403 bp)  1 ICE (397,821 pb) | *alt* | IHQ | PRJEB8966^a^ |
| *A. rivipollensis* | BVH40 | Human stool | 4.70 | 123 | 79 | 105,841 | 61.4 | 4,204 | 16 | 1 IME (19,710bp), 1 ICE (86,889 bp) | - | IHQ | PRJEB9017^a^ |
| *A. rivuli* | DSM 22539^T^ | Freshwater | 4.53 | 102 | 99 | 155,151 | 60.0 | 4,149 | 16 | 1 ICE (132,307 bp) | - | IHQ | PRJEB7035^a^ |
| *A. salmonicida* subsp. *salmonicida* | CIP 103209^T^ | Salmon | 4.74 | 128 | 117 | 89, 543 | 58.5 | 4,442 | 21 | 1 IME w/o identified DR (37458bp) | - | IHQ | PRJEB7036^a^ |
| *A. salmonicida* subsp. *salmonicida* | A449 | Trout | 5.04 | 6 | NA | 5,040,536 | 58.2 | 4,721 | 117 | 0 | - | complete | PRJNA16723^b^ |
| *A. salmonicida* subsp. *salmonicida* | J227 | Infected fish | 4.70 | 122 | 8 | 88,926 | 58.5 | 4,396 | 29 | 1 IME (61,052 bp)  1 ICE (196,895 bp) | - | SD | LSGX01^b^ |
| *A. salmonicida* subsp. *achromogenes* | AS03 | Infected fish | 4.96 | 69 | 21 | 124,543 | 58.3 | 5,074 | 432 | 1 IME (46,013bp) | - | HQ | AMQG02^b^ |
| *A. salmonicida* subsp. *smithia* | JF4097 | Fish | 4.31 | 344 | 89 | 28,949 | 58.7 | 4,411 | 140 | 1 ICE (166,232 bp) | *colAh* | HQ | JZTI01^b^ |
| *A. salmonicida* subsp. *masoucida* | NBRC 13784 | Fish blood | 4.50 | 227 | 92 | 43,858 | 58.8 | 4,193 | 17 | 0 | - | HQ | BAWQ01^b^ |
| *A. salmonicida* subsp. *pectinolytica* | 34mel | River water | 4.77 | 253 | 21 | 47,147 | 58.5 | 4,273 | 35 | 1 ICE (130,872,bp) | - | HQ | ARYZ02^b^ |
| *A. sanarellii* | LMG 24682^T^ | Human wound | 4.19 | 98 | 121 | 82,664 | 63.1 | 3,828 | 1 | 1 IME w/o identified DR (contig 35) (16678bp) | - | IHQ | PRJEB7037^a^ |
| *A. schubertii* | CECT 4240^T^ | Human wound | 4.13 | 111 | 260 | 108,810 | 61.7 | 3,808 | 10 | 0 | - | IHQ | PRJEB7038^a^ |
| *A. simiae* | CIP 107798^T^ | Healthy monkey | 3.99 | 100 | 86 | 73,112 | 61.1 | 3,654 | 21 | 2 IMEs w/o identified DR (24,584 bp and 16,911 bp) | - | IHQ | PRJEB7039^a^ |
| *A. sobria* | CECT 4245^T^ | Fish | 4.68 | 52 | 34 | 188,072 | 58.6 | 4,160 | 24 | 0 | - | IHQ | PRJEB7040^a^ |
| *A. taiwanensis* | LMG 24683^T^ | Human wound | 4.24 | 106 | 66 | 85,294 | 62.8 | 3,884 | 3 | 0 | - | IHQ | PRJEB7041^a^ |
| *A. tecta* | CECT 7082^T^ | Human diarrheic stool | 4.76 | 51 | 8519 | 238,229 | 60.1 | 4,278 | 2 | 1IME (27,586 bp) | - | IHQ | PRJEB7042^a^ |
| *A. veronii* bv. *veronii* | CECT 4257^T^ | Human respiratory tract (drowning) | 4.52 | 52 | 59 | 181,171 | 58.8 | 4,070 | 9 | 0 | - | IHQ | PRJEB7044^a^ |
| *A. veronii* bv. *sobria* | LMG 13067 | Environment | 4.74 | 72 | 46 | 147,470 | 58.3 | 4,171 | 20 | 0 | - | IHQ | PRJEB7051^a^ |
| *A. veronii* | CECT 4486 | Surface water | 4.41 | 66 | 70 | 147,024 | 58.4 | 3,997 | 5 | 0 | - | IHQ | PRJEB7050^a^ |
| *A. veronii* | B565 | Aquaculture pond sediment | 4.55 | 1 | NA | 4,551,783 | 58.7 | 4,073 | 23 | 0 | - | complete | CP002607^b^ |
| *A. veronii* | BVH25b | Human respiratory tract | 4.66 | 35 | 63 | 241,725 | 58.7 | 4,185 | 14 | 0 | - | IHQ | PRJEB9014^a^ |
| *A. veronii* | BVH26b | Human wound | 4.58 | 48 | 73 | 180,501 | 58.7 | 4,107 | 21 | 0 | - | IHQ | PRJEB9015^a^ |
| *A. veronii* | 77c | Human diarrheic stool | 4,61 | 42 | 78 | 230,104 | 58.6 | 4,124 | 12 | 0 | - | IHQ | PRJEB9012^a^ |

a: Performed at the Microbial Analysis, Resources and Services (MARS) facility at the University of Connecticut (Storrs, USA)

b: Obtained from National Center for Biotechnology Information GENOME

c: Predicted from ISfinder (ISsaga) analysis (Mobile Genetic Elements team- CNRS, UMR5100, Toulouse, France)

d: Predicted from ICEfinder analysis (Microbial Bioinformatics Group, Shanghai, China)

Abbreviations: SD: Standard draft; HQ: High quality draft genome; IHQ: Improved high quality draft genome; NA: Not available; w/o: without; DR: Directed repeats; ICE: Integrative and conjugative element; IME: Integrative and mobilizable elements
